# Automaticity and Primacy of Auditory Streaming: Concurrent Subjective and Objective Measures

**DOI:** 10.1037/xhp0000146

**Published:** 2015-09-28

**Authors:** Alexander J. Billig, Robert P. Carlyon

**Affiliations:** 1 MRC Cognition and Brain Sciences Unit, Cambridge, United Kingdom

**Keywords:** perceptual organization, auditory scene analysis, auditory streaming, objective measures, attention

## Abstract

Two experiments used subjective and objective measures to study the automaticity and primacy of auditory streaming. Listeners heard sequences of “ABA–” triplets, where “A” and “B” were tones of different frequencies and “–” was a silent gap. Segregation was more frequently reported, and rhythmically deviant triplets less well detected, for a greater between-tone frequency separation and later in the sequence. In Experiment 1, performing a competing auditory task for the first part of the sequence led to a reduction in subsequent streaming compared to when the tones were attended throughout. This is consistent with focused attention promoting streaming, and/or with attention switches resetting it. However, the proportion of segregated reports increased more rapidly following a switch than at the start of a sequence, indicating that some streaming occurred automatically. Modeling ruled out a simple “covert attention” account of this finding. Experiment 2 required listeners to perform subjective and objective tasks concurrently. It revealed superior performance during integrated compared to segregated reports, beyond that explained by the codependence of the two measures on stimulus parameters. We argue that listeners have limited access to low-level stimulus representations once perceptual organization has occurred, and that subjective and objective streaming measures partly index the same processes.

Perceptual organization is the allocation of sensory information to one or more object representations, based on features extracted over a range of spatial, temporal, and spectral scales. In vision, and to a lesser extent audition, much is now known about the nature of such features and how they are computed (Goldstein, 2010; Moore, 2013; Schnupp, Nelken, & King, 2010; Werner & Chalupa, 2013). However, the processes whereby features are assembled into objects remain incompletely understood (Bizley & Cohen, 2013; DiCarlo, Zoccolan, & Rust, 2012; Kubovy & Van Valkenburg, 2001; Treisman, 1996). This article addresses two questions relating to the construction of temporally extended auditory objects, or “streams.” The first concerns the *automaticity* of stream formation: the extent to which it can occur in the absence of attention. The second relates to the *primacy* of stream formation: whether low-level representations of individual auditory events remain accessible once these events have been allocated to streams. One might expect the brain, as an efficient information processing system, to automate such a fundamental operation, and to avoid redundancy by discarding representations of individual sounds after perceptual organization has occurred (Marr, 1982; Shannon, 1948). However, the evidence on both of these issues is equivocal. In the following we will argue that a complete understanding of the nature of auditory streaming requires a combination of both subjective and objective measures. We then present a brief review of the literature as it pertains to the automaticity and primacy of auditory streaming, followed by two experiments that combined percept reports and a performance-based measure with a simple model.

## Measures of Streaming

In many streaming studies, participants continuously report their percept while a sequence of sounds is presented. For example, a common stimulus consists of a series of tones of frequencies A and B repeating in the pattern ABA–ABA–, where the dash represents a silent gap, and listeners continuously report whether they hear a galloping rhythm, corresponding to an integrated percept, or two isochronous sequences of segregated tones (van Noorden, 1975). This general approach has provided considerable insight into the range of factors influencing streaming (see Moore & Gockel, 2002, 2012, for reviews), and is the most direct way of accessing individual, conscious, perceptual experience (Jack & Roepstorff, 2002). However, subjective reports should be evaluated in the context of the task being performed. For example, when participants are instructed to influence their percept (such as in Pressnitzer & Hupé, 2006), they may alter their decision criterion in order to meet the perceived wish of the experimenter, rather than effect a change at an earlier stage of processing (Orne, 1962). Less conscious shifts in decision criterion may also occur when listeners decide between two perceptual interpretations of an ambiguous stimulus (Green & Swets, 1966).

Objective measures aim to assess perception in a manner that is uncontaminated by changes in response criterion. These include recordings of neural activity thought to vary with perceptual organization (e.g., Carlyon et al., 2010; Cusack, 2005; Gutschalk et al., 2005; Hill, Bishop, & Miller, 2012; Pannese, Herrmann, & Sussman, 2015; Snyder, Alain, & Picton, 2006; Sussman, Ritter, & Vaughan, 1999; Szalárdy, Bõhm, Bendixen, & Winkler, 2013), and tasks that are easier to perform when the sounds have been perceptually grouped in a particular way. For example, judging the relative timing of sounds is harder when stimulus parameters promote their segregation into separate streams, compared to stimuli that typically form a single stream (Billig, Davis, Deeks, Monstrey, & Carlyon, 2013; Bregman & Campbell, 1971; Roberts, Glasberg, & Moore, 2002; Thompson, Carlyon, & Cusack, 2011; Vliegen, Moore, & Oxenham, 1999). Objective measures of streaming are also essential for probing perceptual organization when subjective reports are not possible, such as in newborn infants (Demany, 1982; Winkler, Kushnerenko, et al., 2003) and animals (Itatani & Klump, 2014).

However, despite their advantages, objective measures are also not solely influenced by perceptual organization. For example, the detection of temporal gaps between sounds deteriorates as they are made less perceptually similar, even when only two sounds are presented and when streaming is unlikely to be a factor (Divenyi & Danner, 1977; Lister, Besing, & Koehnke, 2002). Electrophysiological measures, such as the mismatch negativity (MMN), can also be hard to interpret. The MMN is elicited by a sound that deviates from regularities established by a preceding sequence, and has been used as a tool to assess perceptual organization. Stimuli can be constructed whereby the deviant is only detected, and MMN purportedly only elicited, when segregation occurs—such as for an intensity deviant in a stream of otherwise identical tones, interspersed with another stream of tones of randomly varying intensity (e.g., Sussman, Ceponiene, Shestakova, Näätänen, & Winkler, 2001). (Note that for this paradigm, unlike the detection of timing differences between tones, segregation is associated with an *improvement* in the detectability of the deviant.) However, a recent study found that this intensity MMN can be elicited when the physical parameters are such that segregation could, in principle, occur, even when task performance indicates that the sequences are not actually being experienced as segregated (Spielmann, Schröger, Kotz, & Bendixen, 2014). A similar dissociation between perceptual experience and the elicitation of MMN has also been reported for duration deviants (Szalárdy, Winkler, Schröger, Widmann, & Bendixen, 2013).

For both subjective and objective approaches, then, factors other than perceptual organization can affect the dependent measure, and to a degree that may vary from listener to listener. The best evidence for the role of a stimulus feature or cognitive factor on streaming should come from convergent findings across measures, especially when these measures are extracted from the same participants and with the same stimuli (Dolležal, Brechmann, Klump, & Deike, 2014; Micheyl, Hanson, Demany, Shamma, & Oxenham, 2013; Micheyl & Oxenham, 2010; Oberfeld, 2014; Roberts et al., 2002; Spielmann, Schröger, Kotz, Pechmann, & Bendixen, 2013; Sussman, Horváth, Winkler, & Orr, 2007; Szalárdy, Winkler, et al., 2013). We take this approach in Experiment 1 of the present study to investigate the much-debated role of attention in auditory streaming.

## Automaticity of Streaming

The formation of auditory streams is to a large extent consistent with the Gestalt grouping principles of similarity, continuation and proximity. The lower the rate of physical change between sequentially presented sounds, the more likely they are to derive from the same source and to be linked to the same perceptual object (Bregman, 1990; Winkler, Denham, Mill, Bohm, & Bendixen, 2012). For example, in the ABA– sequences described in the previous section, listeners typically report an integrated percept at slow repetition rates, or when the A and B tones are close in frequency (van Noorden, 1975). At greater frequency separations (Δf) and faster rates, the pattern tends to segregate into two isochronous streams, each containing tones of a single frequency. For a large range of parameter values, ABA– sequences are initially heard as integrated, with the probability of segregation increasing over time (the “build-up” of streaming; Anstis & Saida, 1985; Bregman, 1978).

Although streaming depends on physical stimulus parameters, it can be shaped by experience, expectations, and the focus of attention (see Snyder, Gregg, Weintraub, & Alain, 2012 for a review). For example, listeners are more likely to hear an ABA– sequence as segregated if an earlier sequence was perceived that way (Snyder, Carter, Hannon, & Alain, 2009). During more prolonged exposure, perception can alternate between different interpretations every few seconds, and listeners report being able to exert some control over the duration of these phases (Pressnitzer & Hupé, 2006). The streaming of speech sounds depends not only on their spectro-temporal characteristics, but also on whether they form a familiar word (Billig et al., 2013).

An issue of considerable theoretical importance concerns the interaction between streaming and attention. It is well established that we can select an already-formed auditory stream for more detailed neural and perceptual processing, as is the case for visual objects (Broadbent, 1958; Desimone & Duncan, 1995; Shinn-Cunningham, 2008). Importantly, endogenously controlled attention can also affect the perceptual organization of the auditory scene itself. Based on a dichotomy proposed by Neisser (1967) for visual processing, Bregman (1990) distinguished between “primitive” mechanisms of auditory scene analysis, operating automatically based on simple stimulus characteristics, and the attentive, “schema-based” identification of familiar sounds in a mixture. In this framework, streaming based on frequency separation was considered primitive and automatic.

However, Carlyon, Cusack, Foxton, and Robertson (2001) demonstrated that streaming of simple tone sequences could in fact be influenced by attention. In that study, listeners heard a repeated ABA– pure tone pattern in one ear and a series of brief noise bursts in the other. In the critical condition, they spent the first 10 s of each sequence discriminating amplitude modulation patterns in the noise bursts. After the noises stopped they switched their attention to the pure tones. These were typically reported as integrated, with build-up taking place over the next few seconds as if the sequence had just started. This was interpreted as evidence that no streaming occurred during the initial part of the sequence when participants were engaged in a competing auditory task. An alternative explanation is that some streaming did occur in the absence of focused attention, but was reset when attention was switched to the tones from the noises (Cusack, Deeks, Aikman, & Carlyon, 2004). Similar results were obtained by Thompson et al. (2011), using an objective measure of streaming. Further evidence for an effect of attention on streaming of ABA– patterns, at least at intermediate frequency separations, comes from comparing streaming- and attention-related modulations of the evoked response measured by electroencephalography (Snyder et al., 2006) and magnetoencephalography (Gutschalk, Rupp, & Dykstra, 2015). Additionally, the perceptual detectability and neural representation of a repeated target sound against a background of irregularly timed tone pips vary in a manner consistent with attention promoting segregation of more complex scenes (Elhilali, Xiang, Shamma, & Simon, 2009).

However, other behavioral and electrophysiological work suggests that focused attention may not always be necessary for streaming to build up. The data of Cusack, Deeks, Aikman, and Carlyon (2004), who used a paradigm similar to that of Carlyon et al. (2001), show only a partial effect of attention at intermediate to larger frequency separations between the A and B tones in an ABA sequence. Macken, Tremblay, Houghton, Nicholls, and Jones (2003) reported that background sounds affect the serial recall of visually presented items in a manner consistent with their segregating into separate streams, despite being task irrelevant. Pannese et al. (2015) showed that attention to one of three concurrent auditory sequences does not prevent patterns in the other two unattended sequences from being detected, as reflected in spectral power in the neural response at the pattern repetition rate. Additionally, the findings from a number of MMN studies are also consistent with the view that focused attention is not necessary for streaming to occur (Sussman et al., 2007; Sussman, Ritter, & Vaughan, 1998), although the uncertainty regarding the link between MMN and percept, referred to in the previous section, should be borne in mind.

A limiting factor in interpreting these studies as evidence for the automaticity of streaming is the strength of the attentional manipulations. Although a demanding visual memory task can effectively engage central resources and prevent sustained effortful processing of the to-be-ignored sounds (Winkler, Sussman, et al., 2003), at least some attentional capacity is modality specific (Duncan, Martens, & Ward, 1997) and may be available to facilitate streaming. Detecting an occasional change in a single feature of a concurrently presented auditory stimulus does not incur a task-sharing cost in divided attention experiments (Bonnel & Hafter, 1998; Pannese et al., 2015; Sussman et al., 2007) and even the demanding amplitude modulation detection task used by Carlyon and colleagues may not fully engage the attention of all participants at all times (Carlyon et al., 2001; Cusack et al., 2004; Thompson et al., 2011). It is therefore possible that, when an incomplete effect of attention is observed, listeners may not have completely engaged in the competing task. In Experiment 1, we reassess the effect of attention on streaming using subjective and objective measures in the same participants. We then test whether a simple model of covert attention can account for any build-up of streaming in the absence of attention, or whether it is more likely that some streaming can occur automatically. As described in the next section and tested in Experiment 2, further insight comes from a direct examination of the link between subjective and objective measures of streaming.

## Primacy of Auditory Streaming: Access to Low-Level Representations

There are many examples of low-level sensory information being discarded in the formation of higher-level representations. In audition, detail about the phase of frequency modulation is unavailable to listeners tracking a tone through noise (Carlyon, Micheyl, Deeks, & Moore, 2004) and listeners represent environmental sound textures using relatively simple time-averaged statistics, making it harder to distinguish between two instances of the same texture at longer stimulus durations (McDermott, Schemitsch, & Simoncelli, 2013). Similar examples from vision include encoding only the mean size of individual dots or the mean orientation of peripherally presented gratings (Ariely, 2001; Parkes, Lund, Angelucci, Solomon, & Morgan, 2001). However, in both modalities, certain properties of the stimulus can be abstracted away in the derivation of one perceptual attribute, while contributing to a parallel computation. For example, illuminance information contributes to brightness judgments but is discarded when assessing the apparent reflectance of the same objects (Arend & Goldstein, 1990). In hearing, the precedence effect describes how echoes are suppressed when a primary waveform and its reflection arrive at the ear sufficiently close in time. Although the second waveform does not form a separate perceptual object, and may not contribute to localization of the source, it does influence the subjective sound quality in a way that is informative about the reverberance of the environment (Benade, 1990; Fitzpatrick, Kuwada, Kim, Parham, & Batra, 1999). Finally, although the identity of speech sounds is typically perceived categorically (Liberman, Harris, Hoffman, & Griffith, 1957), within-category differences in acoustic structure can impact the speed and ease of lexical access (Andruski, Blumstein, & Burton, 1994; McMurray, Tanenhaus, & Aslin, 2002).

Auditory streaming also involves the transformation of low-level information into a more compact representation. Once the waveforms of an ABA– pattern have been recognized as individual pure tones, these are either integrated into a galloping pattern of triplets, or segregated into separate streams of A and B tones. An important question is whether the listener retains access to representations of the original sound elements after this perceptual organization. The answer may not only provide information on the way the brain encodes auditory information, but also help validate the use of temporal-shift tasks as an index of auditory streaming.

Although judging the relative timing of sounds is harder when stimulus parameters favor segregation than integration (Bregman & Campbell, 1971), the fact that discriminating gaps between isolated pairs of sounds deteriorates as frequency separation increases (Divenyi & Danner, 1977) indicates that this need not be due to streaming. To establish whether detection of temporally shifted B tones is an indicator of perceptual organization per se, it is necessary to compare performance when the same set of sounds is perceived as integrated versus segregated. If listeners have access to representations of stimulus elements as they were prior to any perceptual organization, this cue should be equally useful regardless of percept. In this case, performance should depend only on parameters such as the frequency separation between A and B tones and how long the sequence has been playing. However, if early representations of the individual sound elements are no longer available, indicating the primacy of streaming, performance should indeed depend on the reported perceptual organization. We address this question in Experiment 2 by requiring listeners to simultaneously make subjective judgments and perform an objective task on the same sequence of sounds. This allows us to measure the effect of percept on performance, independently of stimulus parameters.

## Experiment 1

### Rationale and Predictions

In Experiment 1, we obtained subjective and objective measures of streaming from a single group of participants in order to reconcile discrepant findings from previous studies on the automaticity of streaming. We presented participants with ABA– sequences with one of two frequency separations between A and B tones, and with short delays to B tones (deviants) occurring either early in the sequence, toward the middle or near the end. For fully attended ABA– sequences, we expected the detection of deviants to be worse later in the sequence and when the frequency separation between A and B tones was greatest. We predicted that reports of segregation would show a corresponding increase with these stimulus manipulations. However, when participants were engaged in a demanding task on concurrently presented noises for most of the sequence before switching attention to the tones, we expected both measures to indicate less streaming than at the same point when they had attended to the tones throughout. We anticipated that subjective reports and deviant detection following an attention switch would be similar to those at the start of an attended sequence if (a) both measures accurately reflected perceptual organization, (b) either no segregation occurred in the absence of attention, or switches of attention fully reset streaming, and (c) the noise task fully engaged participants’ attention. This last requirement is tested using a simple model. Other patterns of results would indicate that one or more of the assumptions were false.

### Method

#### Participants

Twelve naïve listeners (five males, age range 20–31 years, mean age = 24.5 years) with normal hearing (average pure tone threshold <20 dB HL for 500–3,000 Hz, the frequency range spanned by the stimuli) were recruited from the Medical Research Council Cognition and Brain Sciences Unit participant panel and paid for their time. All experimental procedures were approved by the Cambridge Psychology Research Ethics Committee.

#### Materials

**Figure 1 fig1:**
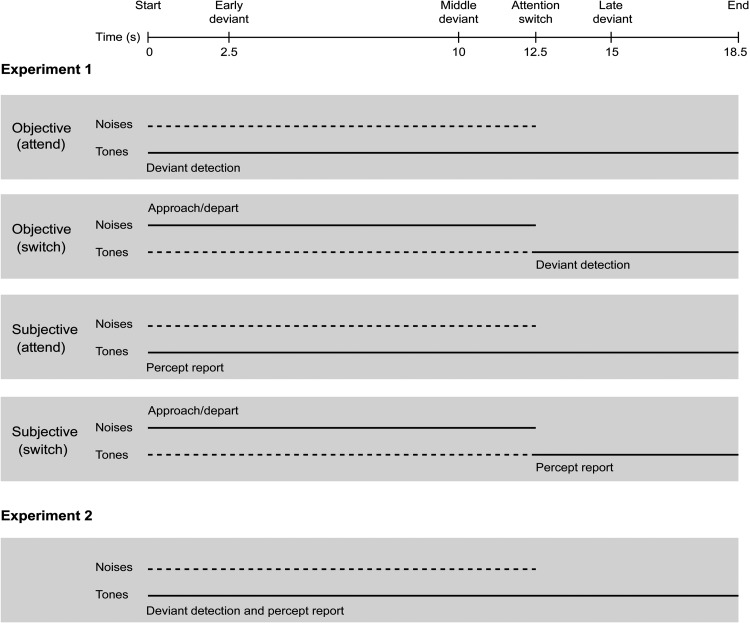
Each panel represents a separate condition: four in Experiment 1 and one in Experiment 2. The attended sounds are shown with solid lines and the ignored sounds with dashed lines. The task for the attended sounds is indicated above/below the corresponding line. The timeline at the top shows the occurrence of potential events, averaged across sequences. However, all of these events, with the exception of the start of the sequences and the early deviants, could occur up to 1 s earlier or later than plotted.

Sequences of 35, 37, or 39 ABA– triplets were presented to one ear, where A and B were 50-ms pure tones including 10-ms linear onset and offset ramps. The frequency of the A tone was drawn on a sequence-by-sequence basis from a log-rectangular distribution centered on 800 Hz and spanning one octave. The B tone frequency for a given sequence was either four or eight semitones higher than the A tone (Δf = 4 or Δf = 8). In the majority of triplets (“standards”), the silent interval was 75 ms between tones of different frequency (i.e., within a triplet) and 200 ms between triplets. In occasional “deviant” triplets the B tone occurred 50 ms later than in the standards. These parameters were chosen to match conditions in Carlyon et al. (2001), Cusack et al. (2004), and Thompson et al. (2011), and were anticipated to generate intermediate levels of streaming for maximal sensitivity of both measures to the attention manipulation. Sequences lasted between 17.5–19.5 s, with triplets presented every 500 ms. As illustrated in Figure 1, deviants could occur in three positions: early (sixth triplet), middle (18th from last triplet), or late (seventh from last triplet). The eight combinations of deviant position (none; early only; middle only; late only; early and middle; early and late; middle and late; early, middle, and late) were equally represented across sequences.[Fig-anchor fig1]

A series of 400-ms noise bursts was presented to the contralateral ear, beginning at the same time as the triplet sequences. The noise bursts were generated by applying a digital brick wall bandpass filter (2,000–3,000 Hz, 60 dB down in stopbands) to white noise. Two different noise tokens were generated, each giving a different impression of motion: The “approach” noise had a 350-ms linear attack ramp and 50-ms linear decay ramp while the duration of these ramps was reversed for the “depart” noise. The noise bursts were presented on average every 1 s, with a jitter of up to ± 250 ms, drawn from a rectangular distribution. Each noise burst was chosen at random to be either approach or depart with equal probability.

Stimuli were generated digitally at a sample rate of 44,100 Hz with 16-bit resolution. Sounds were presented over Sennheiser HD650 headphones at a level of 55 dB SPL to listeners seated in a double-walled sound-insulated room. For half of the participants, the triplets were always presented to the left ear and the noise bursts to the right ear. For the remaining participants, the allocation was reversed.

#### Procedure

The experimental conditions are shown in the first four panels of Figure 1. The experiment consisted of an objective stage (deviant detection) followed by a subjective stage (percept report). This order was chosen to avoid explicitly making participants aware of the two perceptual organizations, and to reduce the likelihood of them trying to influence their percept in order to improve their detection of deviants. In the objective stage, for half of the sequences (“attend” trials) participants were instructed to attend to the triplets throughout (ignoring the noise bursts) and to detect deviants by pressing a key on a computer keyboard as soon as possible. They were told that each sequence could contain any number of deviants (including none), but were not given any information as to when these could occur. For the other half of the sequences in the objective stage (“switch” trials), participants were instructed to attend initially to the noise bursts (ignoring the triplets) and to label each noise burst as approach or depart using two different keys. When the noise bursts finished, halfway between potential middle and late deviants, a prompt on the screen indicated that they were to attend to the remaining 6 s of triplets and to detect deviants as in the attend trials. The objective stage consisted of 96 trials, one for each combination of Δf (four or eight), deviant configuration (eight possible combinations of early, middle, and late deviants being present/absent), task (attend or switch) and sequence length (35, 37, or 39 triplets). Trials were grouped into eight blocks of 12, each containing a mixture of attend and switch trials. Before the experimental trials, participants were trained to discriminate standard from deviant triplets, and approach from depart noise bursts. They practiced both tasks (deviant detection and noise labeling), initially with only the task-relevant sounds in one ear, and then with triplets and noise bursts presented dichotically (as in the main experiment).

The subjective stage was also divided into attend and switch trials. The instructions were the same as in the objective stage, with the exception that when attending to the triplets, participants were to continuously report their percept instead of detecting deviants. They pressed one button (“one stream”) when hearing the integrated triplet pattern and another button (“two streams”) when hearing the triplets segregate into two isochronous sequences, each containing tones of one frequency. Participants were told to make a selection as soon as possible after the sequence began, to make further responses whenever their percept changed, and to adopt a neutral listening approach (i.e., not to try to hear the triplets in any particular way). The distinction between a change in the streaming percept and the physical difference between standards and deviants was also emphasized. The subjective stage consisted of 32 trials, one for each combination of Δf, deviant configuration, and task. Note that the deviant triplets were not relevant to the task in the subjective stage, but were included to keep the stimulus fixed across stages of the experiment. All sequences in the subjective stage comprised 37 triplets, there being no need to vary the length to avoid participants learning the timings of potential deviants. Trials were grouped into two blocks of 12 and one of eight, and each block contained a mixture of attend and switch trials. Before the experimental trials, the concept of streaming was explained to participants using ABA– patterns with Δf of zero, six, and 12 semitones. Participants practiced the percept report task with monaural triplets, and then with triplets and noise bursts presented dichotically.

In both stages, virtual buttons on a computer screen indicated the current selection or response. Buttons corresponding to responses not currently available were dimmed on the display. An asterisk on one side of the screen indicated the ear to which the participant should be attending at that time. Trials were separated by 2 s of silence, with the order of trials completely randomized for each participant. Participants took self-timed breaks of at least 20 s between blocks. The testing session lasted approximately 90 min including audiometry, training, and breaks.

#### Analyses

For deviant detection, a hit was defined as a “yes” response made within 1.147 s of the start of the additional silence characterizing a deviant. This was the latency within which 95% of all responses were made to deviants, pooled across all conditions and participants. Other “yes” responses were scored as false alarms, and labeled as early, middle, or late according to the potential deviant location to which they were closest in time. For example, in sequences consisting of 37 triplets, false alarms occurring in the first 6.25 s were labeled as early, those occurring between 6.25 and 12.5 s were labeled as middle, and those occurring between 12.5 and 18.5 s were labeled as late. The false alarm rate is traditionally defined as the number of “yes” responses to nonsignal trials divided by the total number of nonsignal trials. The denominator of this ratio for a given section of a given sequence was calculated as follows, based on the method of Bendixen and Andersen (2013). First, the duration of the relevant section of the sequence (5.75 s, 6.25 s, or 6.75 s for early and middle false alarms, 6 s for late false alarms) was divided by 1.147 s (the hit threshold) to give a normalized estimate of the number of “trials”; this number was reduced by 1 whenever there was a deviant in that section, to give the number of “nonsignal trials.” The numerator was the number of “yes” responses during intervals that did not contain a deviant. Hit rates and false alarm rates were averaged across all instances of a given Δf, deviant location and task. In cases where the average hit or false alarm rate was 0 or 1, the numerator was adjusted up or down (respectively) by 0.5 to ensure the *z* transforms for calculating sensitivity (*d*′) were well defined.

For streaming build-up in the subjective stage, the reported percept was sampled at the start of each triplet. For each participant, Δf, and task, the proportion of segregated responses was calculated at each time point by dividing the number of “two stream” responses across trials by the number of trials in that condition for which a response had been made at all. This was only defined at time points for which a participant had responded in at least one trial. Perceptual phase durations were transformed to natural logarithms before testing. Unless otherwise stated, statistics are based on two-sided paired *t* tests and repeated-measures analyses of variance (ANOVAs). Degrees of freedom are corrected for nonsphericity (and reported as such) using the Huynh–Feldt method. All tests are reported as significant based on an α value of .05. Effect sizes are given as η_p_^2^ (proportion variance explained after excluding that attributed to other predictors) for ANOVAs, and as Cohen’s *d* for *t* tests.

### Results

#### Deviant detection (objective stage)

**Figure 2 fig2:**
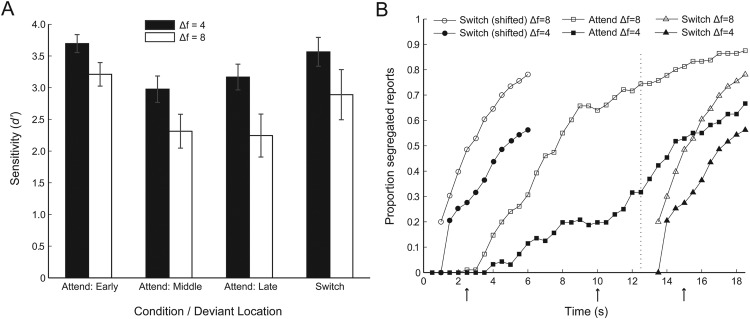
Experiment 1 results. (A) Objective stage: Deviant sensitivity (*d*′) for four-semitone (black bars) and eight-semitone (white bars) frequency separations. There were three deviant positions in attend condition and one the switch condition. Error bars show ±1 *SE* of the mean, with across-participant variance removed. (B) Subjective stage: Proportion of segregated reports for four-semitone (filled symbols) and eight-semitone (empty symbols) frequency separations. Squares indicate reports in the attend condition. Reports in the switch condition are plotted at the actual time they occurred (triangles) and replotted at the start of the sequence (circles) for ease of comparison to the attend condition. The dotted line shows the time at which participants were instructed to switch attention from the noises to the tones in the switch condition. Arrows below the abscissa indicate the mean times at which early, middle and late deviants occurred.

Figure 2A shows that in the attend condition, deviants were best detected at the smaller frequency separation, main effect of Δf on *d*′, *F*(1, 11) = 21.62, *p* < .001, η_p_^2^ = .66, and when they occurred at the start of the sequence, main effect of position on *d*′, *F*(1.35,22) = 21.11, *p* < .001, η_p_^2^= .66; no interaction, *F*(2, 22) = 1.51, *p* = .243, η_p_^2^ = .12. As predicted, performance was poorest when stimulus parameters favored stream segregation. Late deviants were better detected in the switch condition than the attend condition, main effect of task on *d*′, *F*(1, 11) = 6.17, *p* = .030, η_p_^2^= .36; no interaction with Δf, *F*(1, 11) = 1.33, *p* = .274, η_p_^2^ = .11, indicating that the removal of attention from the tones during the first part of the sequence, or the switch of attention itself, improved performance compared to the late attend condition in which the tones were attended throughout. Deviant detection did not differ significantly between attended deviants at the start of the sequence and those that followed a switch of attention, no effect of task/position on *d*′, *F*(1, 11) = 0.66, *p* = .433, η_p_^2^= .06; no interaction with Δf, *F*(1, 11) = 2.06, *p* = .179, η_p_^2^ = .16. The mean percentage of correctly labeled noises in the objective stage was 92% (*SD* = 6%, range 82–98%).[Fig-anchor fig2]

#### Percept reports (subjective stage)

The proportion of segregated responses is plotted in Figure 2B for each frequency separation and as a function of time. The characteristic build-up pattern can be seen during the attend condition (squares), with the probability of segregation increasing over time, and at a faster rate for the greater frequency separation, main effect of Δf, *F*(1, 11) = 32.87, *p* < .001, η_p_^2^ = .75; main effect of time point, *F*(3.13, 11) = 35.24, *p* < .001, η_p_^2^ = .76; interaction, *F*(4.95, 11) = 4.49, *p* = .002, η_p_^2^ = .29. It is also clear that the proportion segregated for the 6 s of the switch condition (triangles) is lower than that occurring during the final 6 s of the attend condition. An ANOVA comparing the switch condition to the end of the attend condition reveals main effects of Δf, *F*(1, 11) = 13.71, *p* = .003, η_p_^2^ = .56, task, *F*(1, 11) = 5.57, *p* = .038, η_p_^2^= .34, and time point, *F*(1.61,11) = 16.49, *p* < .001, η_p_^2^ = .60. Although the figure appears to show greater slopes (faster build-up) in the switch condition than at the end of the attend condition, the Task × Time Point interaction was only marginally significant, *F*(1.81, 11) = 3.36, *p* = .059, η_p_^2^ = .56. In general, these findings reveal a substantial effect of attention on streaming, and parallel those from the objective stage. However, although the proportion of segregated responses in the switch condition was lower than that at the *end* of the attend condition, it was greater than at the *start* of that condition. This comparison is illustrated by the pair of curves at the left of the plot (circles), which show the data for the switch condition replotted so as to start at time zero. It was assessed empirically by entering the first 6 s of the reports from the attend and switch conditions into an ANOVA; this revealed a main effect of task, *F*(1, 11) = 34.64, *p* < .001, η_p_^2^ = .76, alongside the expected main effects of Δf, *F*(1, 11) = 13.91, *p* = .003, η_p_^2^ = .56, and time point, *F*(1, 11) = 24.67, *p* < .001, η_p_^2^ = .69. This stands in contrast to the lack of a significant difference in the objective task (deviant detection) across early attend and late switch conditions. Marginal Δf × Time Point and Δf × Task × Time Point interactions, two-way: *F*(3.00, 11) = 2.77, *p* = .057, η_p_^2^ = .20; three-way: *F*(2.24, 11) = 2.55, *p* = .093, η_p_^2^ = .19, may indicate a tendency for build-up rate to depend on frequency separation at the start of an attended sequence, but less so following a switch of attention.

Note that these analyses only cover responses from 2 s or later after the start of a sequence (or after a switch); for earlier time points, there was at least one participant with no responses in one or more conditions. Additional evidence that the unattended portion of the sequence had some effect on subsequent responses comes from an analysis of each participant’s first report at the start of a fully attended sequence and following a switch. The proportion of “two stream” first reports was greater following a switch than at the start of a fully attended sequence, *F*(1, 11) = 13.47, *p* = .004, η_p_^2^ = .55. Furthermore, the time until the first “two stream” report was shorter (2.7 s) following a switch than at the start of a sequence: 4.3 s; *t*(10) = 4.18, *p* = .002, *d* = 1.93; based only on sequences for which the first segregated report occurred within 6 s, and on the 11 participants with sufficient data collapsed across Δf. The mean percentage of correctly labeled noises in the subjective stage was 94% (*SD* = 3%, range 88–98%).

### Discussion

For fully attended tone sequences, subjective and objective measures of streaming reflected a similar dependence on physical aspects of the sounds, consistent with previous work (e.g., Micheyl & Oxenham, 2010; Thompson et al., 2011). More segregation was reported, and temporal deviants were harder to detect, for the larger frequency separation and later in the sequence. However, the two measures of streaming differed in their sensitivity to the manipulation of attention. In the following discussion, we first interpret the subjective report data, before turning to the apparent discrepancy between these and the deviant detection scores.

When participants performed a competing task on noises presented contralaterally to the tones for the first 12.5 s of a trial, subsequent reports of segregation were reduced compared to when the tones were attended throughout. However, there was more streaming than at corresponding time points at the start of an attended sequence. At face value, this latter finding seems incompatible with streaming requiring focused attention, or being fully reset by an attention switch (cf. Carlyon et al., 2001; Cusack et al., 2004). Before drawing this conclusion, we consider two alternatives. The first relates to the difference in stimulus between the start of the attend condition and following the redirection of attention in the switch condition. Whereas noises were present, but ignored, in the former case, they were absent in the latter case (see Figure 1, third and fourth panels). We believe this is unlikely to account for the different amount of segregation; ignored noises in Carlyon et al. (2001) did not reduce streaming of the same tone sequences compared to a no-noise control condition, even though attending to and switching attention from the same noises affected streaming at least as much as in the current study.

A second possibility is that any streaming in the absence of focused attention was fully reset by an attention switch, but that in some switch trials participants started attending to the tones earlier than instructed. If attention remained fully on the tones until the end of these sequences, any resulting streaming would not have been reset at the 12.5-s mark and would have contributed to the greater than expected levels of segregation. To examine whether such a scenario could account for our data, we created a model in which there is a fixed probability, *q*, that attention is allocated to the tones instead of the noises, in any given 0.5-s time window. The model assumes that attention is fully allocated to either the tones or the noises at any moment, with the object of attention being independently determined for each window. For a given value of *q*, we calculated the expected proportion of sequences for which attention would be continuously allocated to the tones from each time point, and modeled the build-up curve for the switch condition by combining different sections of the observed attend build-up curve in the corresponding proportions. This was applied to the group data, separately for each frequency separation. We then used a one-dimensional statistical parametric map (Pataky, 2012) to test for temporal clusters where the modeled and observed switch data differed significantly. This approach uses random field theory (Adler, 1981; Friston, Worsley, Frackowiak, Mazziotta, & Evans, 1994) to take into account the smoothness of the data in controlling for multiple comparisons. The modeled and observed build-up curves always differed significantly, except for values of *q* between .905 and .998. Such a large probability of attending to the tones in any given 0.5-s window prior to 12.5 s is implausible, given that participants were instructed to attend to the noises and that performance in the noise-labeling task was so high. The subjective report data therefore provide evidence against a simple account whereby attention is, at any one time, exclusively allocated to either the tones or the noises, and where either no streaming occurs in the absence of focused attention, or where streaming is fully reset when attention is directed to the tones. We consider alternatives in the General Discussion.

Given this finding, it was perhaps surprising that in the objective task participants were not significantly worse at detecting deviants after a switch of attention than at the start of an attended sequence. However, this may have been due to a ceiling effect; more than a third of early attend and switch *d*′ scores were within 0.5 of the theoretical maximum (*d*′ = 4.4). In addition, the difference between the early attend and switch conditions, expressed as effect size, did not differ significantly between the two measures (90% confidence intervals [CIs] around η_p_^2^ were 90% CI [.00, .32] for objective measure and 90% CI [.20, .74] for subjective measure). On balance then, reports of segregation and deviant detection performance are broadly consistent (but inversely related) in terms of the effects of physical parameters and attention. However, an evaluation of the true link between streaming measures requires a comparison of deviant detection performance across reports of integration and segregation for identical stimulus conditions. In Experiment 2, subjective and objective measures were collected at the same time to facilitate such a comparison.

## Experiment 2

### Rationale and Predictions

In Experiment 1, percept reports and the detection of temporal deviants were both affected by stimulus parameters in a manner consistent with it being harder to judge the relative timing of sounds that fall in two separate streams than in a single stream. In Experiment 2, the same participants attended to the sequences throughout and performed subjective and objective tasks concurrently. This approach allowed us to compare deviant detection across integrated and segregated percepts and to disentangle the effect of stimulus parameters from that of perceptual organization. If detection of a temporal shift does truly depend on percept, subjective reports should predict performance over and above any effect of frequency separation and the point in the sequence at which the deviant occurred. If, on the other hand, listeners have parallel access to individual stimulus representations independently of how they are organized into streams, performance should depend only on the physical characteristics. This would indicate a limit to the primacy of streaming.

### Method

#### Participants

The same participants as in Experiment 1 took part and were paid for their time. All experimental procedures were approved by the Cambridge Psychology Research Ethics Committee.

#### Materials

The stimuli, and their allocation to left and right ears across participants, were the same as in Experiment 1.

#### Procedure

Testing took place on the same day as Experiment 1, after a short break. Participants were told to ignore the noise bursts and to attend to the triplets (see Figure 1, bottom panel). In all trials, the task was to detect deviants (as in the objective stage of Experiment 1), while continuously reporting the experienced percept (as in the subjective stage of Experiment 1). Participants were instructed to treat the two tasks with equal priority. The experiment consisted of 48 trials, one for each combination of Δf, deviant configuration and sequence length. Trials were grouped into four blocks of 12. Before the experimental trials, participants practiced performing both tasks on the triplet sequences simultaneously, first without and then with noise bursts in the contralateral ear. Virtual buttons on a computer screen indicated the current selection and response. Trials were separated by 2 s of silence, with the order of trials completely randomized for each participant. Participants took self-timed breaks of at least 20 s between blocks. The testing session lasted approximately 30 min including training and breaks.

#### Analyses

Deviant detection and streaming build-up were analyzed with respect to stimulus characteristics as in Experiment 1. Additional analysis steps will be introduced as required.

### Results

#### Deviant detection as a function of stimulus parameters and task demands

**Figure 3 fig3:**
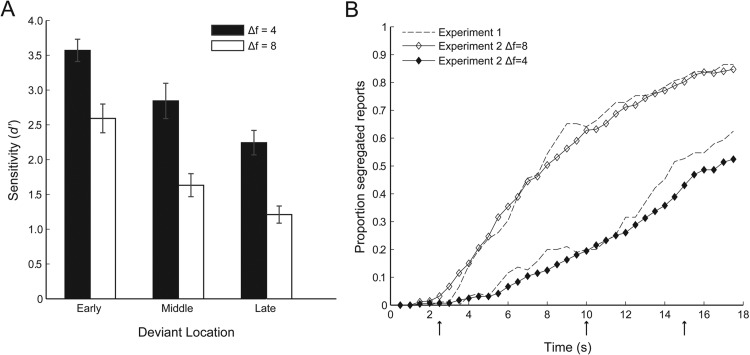
Experiment 2 results. (A) Deviant sensitivity (*d*′) for four-semitone (black bars) and eight-semitone (white bars) frequency separations. Three deviant positions are shown. Error bars show ±1 *SE* of the mean, with across-participant variance. (B) Proportion of segregated reports for four-semitone (filled symbols) and eight-semitone (empty symbols) frequency separations. Results from the attend condition in Experiment 1 are plotted with dashed lines for comparison. Arrows below the abscissa indicate the mean times at which early, middle and late deviants occurred.

Figure 3A shows the results of the deviant detection task in Experiment 2. As in Experiment 1, sensitivity to deviants was greater for the smaller frequency separation, main effect of Δf, *F*(1, 11) = 59.15, *p* < .001, η_p_^2^ = .84, and declined as the sequence progressed, main effect of position, *F*(2, 11) = 27.30, *p* < .001, η_p_^2^ = .71. To establish whether there was a dual task cost for deviant detection we compared sensitivity across experiments. Deviants were less easily detected in Experiment 2, *F*(1, 11) = 12.10, *p* = .005, η_p_^2^= .52, with a mean drop in *d*′ of 0.59. This decrement was greatest at the larger frequency separation, Δf × Experiment interaction, *F*(1, 11) = 5.94, *p* = .033, η_p_^2^ = .35, and for late deviants, Position × Experiment interaction, *F*(1, 11) = 4.42, *p* = .024, η_p_^2^ = .29.[Fig-anchor fig3]

#### Percept reports as a function of stimulus parameters and task demands

As in Experiment 1, the probability of segregation increased over the course of a sequence, and at a faster rate for the greater frequency separation, main effect of Δf, *F*(1, 11) = 88.06, *p* < .001, η_p_^2^ = .89; main effect of time point, *F*(2.63,11) = 89.23, *p* < .001, η_p_^2^ = .89; interaction, *F*(3.24,11) = 15.04, *p* < .001, η_p_^2^ = .58. As can be seen from Figure 3B, the probability of segregation did not differ across experiments, *F*(1, 11) = 1.29, *p* = .279, η_p_^2^ = .11. The smoother build-up curves in Experiment 2 are likely to be due to the average being calculated over a greater number of trials (24 per participant per Δf) than in Experiment 1 (eight per participant per Δf). Figure 3B also illustrates that there is no evidence for the deviants themselves causing streaming to reset; there are no drops in the proportion of segregated reports following the occurrence of deviants at 2.5 s, 10 s or 15 s (indicated by arrows under the abscissa). It is also worth noting that the overall proportion of segregation did not differ significantly as a function the number of deviants (0, 1, 2, or 3) in a sequence, Experiment 1: *F*(1.98, 33) = 0.14, *p* = .864, η_p_^2^ = .01; Experiment 2: *F*(1.16, 33) = 1.01, *p* = .346, η_p_^2^ = .08.

#### Deviant detection as a function of percept report

Sensitivity to deviants was higher when participants reported integration than when they reported segregation, collapsing across Δf and position; *t*(11) = 4.15, *p* = .002, *d* = 1.80. However, we have also shown that percept and sensitivity covary with Δf and position. To establish whether there is an effect of percept on deviant detection independent of stimulus parameters, we compare a series of logistic mixed effects models of the hit/miss data. Modeling the responses to the deviants but not to the standards is justified: participants were more conservative (less likely to report hearing a temporal shift, regardless of whether the stimulus was a deviant) during segregated phases than during integrated phases, *t*(11) = 8.93, *p* < .001, *d* = 2.93. This indicates that differences in hits, not false alarms, drive the sensitivity effect reported earlier.

We used the *glmer* function in *R* package *lme4* (Bates, Maechler, Bolker, & Walker, 2015) to model individual hits and misses for all participants and all conditions. The baseline model contained only a random effect of subject, accounting for variability in overall performance across the group. Progressively more complex models were then fitted by including first fixed effects and then random slopes for Δf, position, and percept, and their interactions. We retained only effects that significantly improved the log likelihood over the previous model, as determined by a chi-squared test with the appropriate number of additional degrees of freedom. The winning model retained fixed effects of Δf, position, and percept, with no interactions (see Table 1 for details). The lack of random slopes for Δf, position, and percept in this model indicates that allowing the strength of these effects to vary by subject was not justified by the data.[Table-anchor tbl1]

Importantly, the inclusion of percept as a factor significantly improved the Δf and position-only model, χ^2^(1) = 77.40, *p* < .001, thereby demonstrating an effect of percept, per se. This was independent of the order in which the effects were added. Given that only 2% of early deviants occurred during a segregated percept compared to 41% of middle deviants and 64% of late deviants (across participants and Δf conditions), we repeated the process excluding the early deviants. The result was the same, with percept significantly improving the Δf and position-only model, χ^2^(1) = 73.47, *p* < .001.

Due to our use of variable sequence lengths, middle and late deviants did not occur a fixed time after the beginning of the sequence, but could vary by up to 2 s within each category (middle deviants: 9, 10, or 11 s into sequence; late deviants: 14, 15, or 16 s into sequence). To check whether the effect of percept could be explained by its covariation with deviant subposition, we tested whether sensitivity depended on subposition, separately for each Δf and position (middle or late). There was no significant effect of subposition on sensitivity in any condition, Δf = 4, position = middle, *F*(2, 11) = 0.27, *p* = .766, η_p_^2^ = .02; Δf = 4, position = late, *F*(2, 11) = 0.68, *p* = .516, η_p_^2^ = .06; Δf = 8, position = middle, *F*(2, 11) = 0.70, *p* = .503, η_p_^2^ = .06; Δf = 8, position = late, *F*(2, 11) = 0.46, *p* = .637, η_p_^2^ = .04. An additional analysis (not shown) ruled out the possibility that the observed effect of percept could be attributed to listeners having difficulty in detecting a deviant shortly after making a change-of-percept report.

We quantify the predictive power of the model using the adjusted count pseudo-*R*^2^ (Long, 1997). Fitted logistic models predict values on a continuum from 0 (definite miss) to 1 (definite hit), which we quantize, declaring a hit for values greater than 0.5 and a miss for values less than 0.5. This allows us to calculate the proportion of outcomes our winning model correctly predicts, over and above those successfully fitted by a simple one that always picks the most frequent outcome across the whole group. This adjusted count pseudo-*R*^2^ value therefore lies between 0 (no additional variance explained) to 1 (all additional variance explained). The model for the middle and late deviants based on Δf and position has an adjusted count pseudo-*R*^2^ value of .22. Including percept increases this to .36. This additional 14% variance explained may be an underestimate of the true effect of percept, as the effects of frequency separation and deviant position might themselves be mediated by percept.

### Discussion

Previous work (Dolležal et al., 2014; Micheyl & Oxenham, 2010; Spielmann et al., 2013) has indicated that subjective and objective behavioral measures of pure tone streaming correlate across stimulus conditions at a group level. The present study goes further, showing that deviant detection was significantly worse when listeners reported hearing a segregated percept, independent of frequency separation, the position of the deviant in the sequence and any dual-task interference effects. Although subjective reports and neural measures of streaming have often been collected simultaneously (Cusack, 2005; Dykstra et al., 2011; Gutschalk et al., 2005; Hill et al., 2012; Snyder et al., 2006; Szalárdy, Bõhm, et al., 2013; Szalárdy, Winkler, et al., 2013), to our knowledge, only one previous study has directly linked an objective behavioral measure of streaming with concurrent percept reports. Participants in Billig et al. (2013) heard sequences of repeated syllables that could be perceived as integrated or segregated due to spectral differences between the initial /s/ sound and the remainder (such as “stone” vs. “s” + “dohne”). Gaps occasionally inserted between the two parts of the syllable were easiest to detect when listeners reported hearing a single syllable compared to when they reported two separate sounds. That finding was obtained in the context of speech sounds, for which streaming is affected by nonsensory factors such as lexicality; our results demonstrate that this relationship also holds for the pure tones patterns more commonly used to study streaming.

Experiment 2 also reveals that not all variance in one measure can be explained by the other. For example, just 2.5 s after the start of a sequence, listeners were significantly more sensitive to deviants when the tones were separated by four than by eight semitones, *F*(1, 11) = 5.38, *p* = .041, η_p_^2^ = .32. In contrast, subjective reports revealed almost no streaming in either condition at this time point (see Figure 3B). This is line with previous accounts that increased frequency separation between a single pair of tones impairs gap discrimination regardless of streaming (Divenyi & Danner, 1977). Additionally, average performance was far above chance (*d*′ = 2) in the poorest condition (late deviants, Δf = 8) when segregation was reported to occur 80% of the time. In this case participants may have made use of a secondary cue, the 10% change in inter onset interval between a deviant B tone and the previous or succeeding B tone, compared to across standard triplets. Gap duration difference limens at 125 ms and 500 ms inter onset intervals are such that this cue is much weaker than that provided by the 40% change in the interval between the B tone and the previous or succeeding A tone (Friberg & Sundberg, 1995). Nonetheless, Oberfeld (2014) showed that it can contribute somewhat to gap discrimination performance under stimulus conditions favoring segregation.

An unresolved question is why it should be harder to compare the timing of two sounds when they fall in separate streams compared to a single stream, stimulus differences aside. One explanation is that it may not be possible to attend simultaneously to two streams, or to switch attention sufficiently quickly, in order to *extract* timing information from both (Bregman & Campbell, 1971). Alternatively, a failure to *compare* timing information could arise if onsets are encoded with respect to a stream-specific temporal frame of reference, rather than relative to a global clock. If this were the case, calculating the interval between sounds falling in separate streams would require the additional step of converting onset times to a common reference, incurring a processing cost not encountered when the sounds fall in a single stream. Such a coding scheme would fit well with theoretical and experimental work in vision on the hierarchical representation of position. Watt (1988) proposed that a scene is initially parsed into groups of neighboring elements. The position of each element is then encoded relative to the group to which it belongs, while the position of each group is stored relative to others. In such a scheme, the relative position of elements across groups is represented only indirectly. Baylis and Driver (1993) argued that this hierarchical position coding fitted well with object-based theories of visual attention (Duncan, 1984) and could account for data from an experiment conceptually similar to ours. In that study, participants made a speeded judgment of the relative height of two apices in the outlines of solid shapes (Baylis & Driver, 1993). The stimulus was such that the apices could be perceived as both belonging to a single central object, or as each belonging to a separate object on either side of the display. Participants who had been primed to see a single object were better able to judge the relative height of the two apices than those primed to see two objects. The parallel with our Experiment 2 is clear: The position of the apices is analogous to the timing of the A and B sounds, and the perception of one/two visual objects corresponds to experiencing integration/segregation. In both cases, an across-object (across-stream) comparison would be problematic if the features to be judged were encoded with respect to local rather than global reference frames.

A comparison of Experiments 1 and 2 showed that performing both tasks simultaneously led to a moderate decrement in deviant detection, which was more pronounced later in the sequence and at the greater frequency separation, that is, when the stimulus parameters favored streaming. Our paradigm allows us to discount one possible explanation, which is that asking participants to report their percept led to an amplification of the effects of frequency separation and build-up on streaming; the dynamics of the reports did not differ across the two experiments (Figure 3B). An alternative is that the cognitive load arising from the concurrent performance of two tasks increased later in the sequence, and in particular when deviant detection required a comparison of more physically distinct sounds. This account would provide a further example of how deviant detection and subjective reports can diverge, this time in their sensitivity to interference from a concurrent task.

## General Discussion

### Automaticity of Streaming

In the first experiment, subjective reports and deviant detection performance showed that switching attention to a tone sequence after performing a competing auditory task reduces the likelihood of the sequence being heard as segregated, compared with when that sequence had been attended throughout. This observation is in line with previous findings using subjective reports (Carlyon et al., 2001; Cusack et al., 2004) and objective measures (Thompson et al., 2011). Whether the reduction in streaming is caused by the withdrawal of attention or by its subsequent allocation to the tones cannot easily be addressed by behavioral studies such as these.

Our results are also compatible with other behavioral and electrophysiological data suggesting some streaming can occur in the absence of focused attention (e.g., Macken et al., 2003; Sussman, Bregman, Wang, & Khan, 2005; Sussman et al., 2007) and parallel the finding in vision that perceptual alternations slow but do not completely cease when observers orient away from multistable images (Pastukhov & Braun, 2007). In contrast to Carlyon et al. (2001), participants in the present study reported significantly more segregation following a switch of attention than they did at the start of a sequence; this was the case at both smaller (four-semitone) and larger (eight-semitone) frequency separations. Cusack et al. (2004) observed such a difference at eight- and 10-semitone separations only, but close inspection of their data and those of Carlyon et al. (2001) also reveal numerical differences in the same direction for smaller frequency separations. The collective picture based on subjective reports across these studies, then, is that pure tone patterns can stream apart to some extent, even when attention is diverted away from them sufficiently to result in high performance on a competing auditory task. Furthermore, switching attention to the tone pattern after several seconds may reset this streaming, but not to a state mirroring that at the beginning of a sequence. One of the novel contributions of the present study, arising from our modeling of the subjective data in Experiment 1, is to argue against a simple account whereby the apparent build-up and partial reset of streaming is an artifact of participants’ attention sometimes being exclusively allocated to the tones earlier than instructed.

### Partial Resetting of Streaming

The concept of “partial resetting” requires some explanation. At any given time, a participant experiences either integration or segregation—reports of these percepts are averaged over trials and participants for statistical analysis and for visual representation of the overall proportion of segregation. Our observation is that this proportion is lower in the first few seconds following a switch of attention than at the corresponding time points when the sequence is attended throughout, but higher than at the corresponding time points at the start of the trial. We cannot say very much about how an individual’s percept changes at the moment of the switch because their subjective experience is unknown prior to any reports being made (both when attending to the competing task and immediately following the switch). Nonetheless, it is clear from Figure 2B that although the first report after a switch is rarely of segregation, the proportion of segregated responses begins to increase earlier than at the start of a trial. By the end of the sequence these approach the proportion observed when the sequence is attended throughout. In sum, switching attention to the tones is likely to end any currently experienced segregated percept, but the subsequent integrated phase will in general be shorter than that experienced at the beginning of a sequence. Some perceptual memory may remain for the time-averaged proportion of streaming prior to the switch, a proportion to which the system returns given enough time. A comparison of panels 1 and 3 in Figure 10 of Cusack et al. (2004), which used similar stimulus parameters as the present study, indicates that such a return process may be complete by about 8–10 s after an attention switch.

Cusack et al. (2004) suggested that starting to attend to a set of sounds may force them to form a single perceptual object, whether attention is deliberately switched from another stimulus or exogenously drawn following silence. The authors based this link between the two situations on the fact that, in their data, both led to a near-complete resetting of streaming. We believe this account is still feasible for both types of resetting but that there is some perceptual memory for the previous organization that may cause subsequent segregation of the same pattern to occur more rapidly. Although Cusack et al. (2004) proposed that perceptual history is disregarded following a silent gap, their data do show a marginally significant effect of the length of the gap (1–10 s) on subsequent streaming. The possibility that the decay of segregation during silence takes some finite time, rather than being immediate, is supported by findings from other groups. For example, Denham, Gyimesi, Stefanics, and Winkler (2010) reported that integrated phases following silent gaps of 200–500 ms were shorter than those experienced at the start of sequences. In another study using repeating AAB tone sequences, within-stream intensity deviants continued to elicit MMN shortly after a 3.75 s silent interruption (Sussman-Fort & Sussman, 2014). Furthermore, Snyder and Weintraub (2013) showed that the percept reported at the end of one sequence predicted the amount of segregation in a sequence beginning more than 8 s later. A corresponding finding from vision research is that when an ambiguous visual stimulus is interrupted for several seconds and subsequently restored, the percept typically remains the same (Adams, 1954; Leopold, Wilke, Maier, & Logothetis, 2002; Orbach, Ehrlich, & Heath, 1963; Pastukhov & Braun, 2013; Pearson & Brascamp, 2008).

We suggest, then, that partial resetting in the proportion of segregated reports over trials and participants reflects two phenomena: first, that the immediate percept following a silent gap or switch of attention is likely to be of integration; second, that the prevalence of streaming prior to the interruption or change of attentional focus has a bearing on the subsequent perceptual dynamics.

### Listening Strategies

This account of partial resetting is based largely on subjective report data. However, the deviant detection results in Experiment 1 might be considered more compatible with attention resetting streaming completely rather than partially (Thompson et al., 2011). Here, we consider an account for this apparent difference based on different listening strategies being employed across the two stages of the experiment. During debrief, most participants reported that deviant detection was easier during integrated phases. Although instructed to listen neutrally, they may have succeeded in promoting integration in the objective stage of Experiment 1 to improve deviant detection (Billig, Davis, & Carlyon, 2015; Pressnitzer & Hupé, 2006; van Noorden, 1975). In the subjective stage of that experiment, there was no incentive to promote integration so participants might have been expected to adopt a more neutral listening approach. A similar account was offered by Roberts, Glasberg, and Moore (2008) to explain a discrepancy between their objective findings regarding stimulus changes that reset streaming, and subjective reports from Rogers and Bregman (1993, 1998). However, this explanation seems unlikely to account for our data. In Experiment 2, participants had the same incentive to integrate the sounds as in the objective stage of Experiment 1, but Figure 3B shows that reports of segregation were similar across the two experiments. Furthermore, we have shown that the subjective and objective measures did not differ significantly in terms of the resetting effect size, and that ceiling deviant detection may have obscured a partial resetting effect. Experiment 2 also provided strong evidence that the two measures partly index the same processes.

### Primacy of Streaming

Whereas the pitch and intensity of a sound can be evaluated as high or low in isolation, onset time is defined only in relation to other events. The accuracy with which a sound’s temporal characteristics can be judged therefore depends on exactly which intervals are represented in perception and memory. Our results indicate that streaming limits the form of these representations, or their accessibility. Had deviant detection depended only on stimulus parameters, but not on percept, this would have indicated that listeners could always judge the onset of each B tone against the event most likely to maximize task performance, namely the onset of one of the neighboring A tones. A novel element of our design critical to establishing this primacy of streaming was the collection of subjective reports while participants were detecting deviants, allowing comparison of performance across percept reports for physically identical sounds presented at the same point in a sequence.

It is important to point out the primacy of streaming that we propose does not contradict evidence suggesting that listeners can exercise some control over their percept (Billig et al., 2015; Pressnitzer & Hupé, 2006; van Noorden, 1975), nor is it simply a reformulation of the statement that such control is limited (Thompson et al., 2011). Participants may indeed have been able to influence their percept, resulting in a greater proportion of “integrated” reports than if they had listened neutrally. The point is rather that they did not have access to the output of an earlier or parallel computation of temporal intervals, carried out automatically on representations that were independent of the consciously experienced percept.

The loss of temporal information relating pairs of tones is one of many examples of low level representations being inaccessible once more abstract models of sensory information have formed (Ariely, 2001; Carlyon et al., 2004; McDermott et al., 2013; Parkes et al., 2001). However, our results contrast with a recent finding by Liu, Tsunada, Gold, and Cohen (2015), who also examined the effect of perceptual organization on access to low-level sensory representations. Participants in that study heard a rapid series of tones and had to decide whether they increased or decreased in frequency. The interval between the tones was manipulated such that they were perceived as a single gliding sound (for short intervals) or as discrete sounds (for longer intervals). Despite this effect of interval length on perception, the efficiency with which participants accumulated evidence to form a decision about the mean direction of frequency change did not depend on the presentation rate. This suggests that the primacy of a perceptual abstraction may depend on the type of organization (fusion of pips into a single tone vs. integration of tones into a single stream), the feature of the low-level representation to be accessed (frequency vs. timing), or other task demands (cf. reverse hierarchy theory; Hochstein & Ahissar, 2002; Nahum, Nelken, & Ahissar, 2008). Systematic manipulations of these variables in future work could shed light on the circumstances under which the brain retains, limits conscious access to, or discards different types of sensory information.

### Summary

Although auditory stream segregation is facilitated by focused attention, it can in some circumstances, and to some extent, occur automatically. Switches of attention tend to reset perception to integration but streaming builds up again more rapidly than at the start of a sequence, indicating that refocusing attention, like a silent gap, does not remove all trace of earlier perceptual experience. The detection of occasional temporal shifts in the B tone of an ABA– sequence is easier when the pattern is heard as integrated in a single stream, regardless of the frequency difference between the tones and the time in the sequence at which the deviant occurs. As well as validating the use of this common objective measure of streaming, our finding demonstrates the primacy of streaming. Once sounds have been organized into separate auditory streams, listeners are less able to access low-level representations of the individual events and the temporal relationships between them.

## Figures and Tables

**Table 1 tbl1:** Details of Winning Logistic Regression Model for Middle and Late Hits and Misses in Experiment 2

Effect	*z*-score	*df*^b^	Cumulative log likelihood	*p*^c^	Cumulative adjusted count *R*^2^
Subject (random)	—	—	−365.50	—	.04
Δf (fixed)	5.68	1	−324.53	<.001	.22
Position (fixed)	2.06	1	−315.77	<.001	.22
Percept (fixed)	7.06	1	−279.03	<.001	.36
^a^ *z*-Score for effect based on parameter estimate and standard error (not shown). ^b^ Additional degrees of freedom associated with effect. ^c^ Probability of increase in model log likelihood of at least this magnitude under null hypothesis (χ^2^ test).
